# Research priorities on ending child marriage and supporting married girls

**DOI:** 10.1186/s12978-015-0060-5

**Published:** 2015-09-03

**Authors:** Joar Svanemyr, Venkatraman Chandra-Mouli, Anita Raj, Ellen Travers, Lakshmi Sundaram

**Affiliations:** Department of Reproductive Health and Research, World Health Organization, 20 Avenue Appia, 1211 Geneva 27, Switzerland; Department of Reproductive Health and Research, Adolescent Sexual and Reproductive Health, World Health Organization, 20 Avenue Appia, 1211 Geneva 27, Switzerland; University of California San Diego, Center on Gender Equity and Health, 9500 Gilman Drive #0507, La Jolla, CA 92093-0507 USA; Girls Not Brides, Brook Green, First Floor, Building 5 50 Brook Green, London, W6 7BJ UK

## Abstract

Over the past few years the issue of child marriage has received growing political and programmatic attention. In spite of some progress in a number of countries, global rates have not declined over the past decade. Knowledge gaps remain in understanding trends, drivers and approaches to ending child marriage, especially to understand what is needed to achieve results on a large scale. This commentary summarizes the outcomes of an Expert Group Meeting organized by World Health Organization to discuss research priorities on Ending Child Marriage and Supporting Married Girls. It presents research gaps and recommends priorities for research in five key areas; (i) prevalence and trends of child marriage; (ii) causes of child marriage (iii) consequences of child marriage; (iv) efforts to prevent child marriage; (v) efforts to support married girls.

## Introduction

More than 700 million women alive today were married before their 18th birthday [1]. Child marriage is both a grave violation of human rights and a stumbling block to achieving development goals related to gender, health and education [2–7]. Progress in reducing rates of child marriage is being made in a number of countries, particularly among younger adolescents [3]. Yet global rates of child marriage remain alarmingly high and require strengthened policy and programmatic efforts informed by strong evidence of what works.

While there has been growing recognition of the scale and impact of child marriage and increasing investment to address it the past few years [2, 3], knowledge gaps remain, particularly around effective approaches to addressing child marriage at scale. To address these gaps in knowledge and to identify research priorities on ending child marriage and supporting married girls, WHO organized an expert group meeting in December 2013 in collaboration with Girls Not Brides and UNICEF. Meeting participants included leading researchers and academics, international and national non-government organizations, development agencies, private foundations and United Nations agencies. Participants discussed existing evidence, research gaps and potential priorities in relation to five key areas; (i) prevalence and trends of child marriage; (ii) causes of child marriage (iii) consequences of child marriage; (iv) efforts to prevent child marriage; (v) efforts to support married girls. The issue of understanding child marriage in the broader context was also discussed. This article provides a summary of the discussions and some of the main recommendations.

### Research and evidence gaps on prevalence and trends of child marriage

Child marriage is a global issue but rates vary dramatically, both within and between countries. While there are significant data on prevalence and trends of child marriage across countries, a range of issues require further exploration. Our understanding of intra- and inter-country differences in child marriage is limited and more segmented analyses are needed that cover not only geographic variety, but religion, ethnicity, education, social class and so on [8].

**Table:** 

Recommendations for further research	
•	More age disaggregated data, particularly for the age group 10–14.
•	Trends in age of marriage in relation to other relevant indicators (e.g. health, agency, education, economic empowerment, employment, violence, food security / nutrition, mobility indicators).

### Research and evidence gaps on causes of child marriage

Studies from Africa and South Asia point to the root causes and exacerbating factors that contribute to child marriage - traditions and gender-discriminatory norms rooted in patriarchal values and ideologies, the lack of educational and economic alternatives to child marriage, as well as exacerbating social factors such as poverty, economic instability and conflict and humanitarian crisis [9, 10].

These factors require further examination, particularly in light of evolving social, political, economic and environmental factors, e.g. increasing trends of urbanization [11], improved access to education [12], climate-change related droughts and related food insecurity in the Sahel [13], migration within and between countries, changes – both positive and negative - in labour markets, and civil strife and war. For studying these complex inter-connected issues, new research methods and tools will need to be developed. While the focus on Africa and South Asia is important because of the large numbers of girls involved, other regions where child marriage rates are increasing – Central Asia, North Africa and Latin America - also need attention.

Further, there is very little research on the perceived benefits and costs of child marriage and of delaying marriage, nor is there research on normative shifts in expectations of marriage (e.g., transactional aspects of marriage, changing trends in households from extended to nuclear families, and about relationships (e.g., acceptability of dating and period of engagement) affecting child marriage. We also know little about resiliency factors that impede the practice of child marriage within population groups and geographic areas disproportionately affected.

**Table:** 

Recommendations for further research	
•	Changes in factors contributing to child marriage in well studied areas e.g. Africa and South Asia, and great attention to less studied areas e.g. Central Asia, North Africa and Latin America.
•	Impact of structural factors such as urbanization, migration, climate change and resultant food insecurity, changes in labour markets, civil strife and war on child marriage.
•	Better understanding of the normative shifts in perceptions of and expectations of marriage.
•	Protective factors which impede child marriage in areas where the practice is prevalent, including positive deviants.

### Research and evidence gaps on consequences of child marriage

It is well documented that child marriage leads to a range of negative reproductive health outcomes [10, 14–19]. Less studied are other health consequences such as mental health and malnutrition. Studies from Ethiopia, Afghanistan and the Kurdistan Region of Iraq, point to links between child marriage and suicide and self-immolation respectively [20–22].

Differences in vulnerabilities between younger and older adolescents are not a focus of most of the work in this field and require further research. Although girls under the age of 15 constitute 2 million of the 7.3 million births to underage (<18 years) girls that occurs annually, and these girls face greater risks from child marriage and adolescent childbirth, they are not well represented in the research [10, 23, 24].

Similarly, research has tended to focus on the short-term impact of child marriage [6]. We need evidence on the long-term and intergenerational effects of child marriage. This includes economic costs of early marriage.

**Table:** 

Recommendations for further research	
•	Health and social consequences of child marriage, beyond maternal and perinatal health.
•	Special health and social vulnerabilities of younger adolescent girls.
•	Longitudinal data on the intergenerational impact of child marriage and its relationship to social, development, health and gender issues.
•	Data on the economic costs of child marriage including early childbearing, maternal morbidity and mortality, abortion, violence, and decreased educational and employment potential.

### Research and evidence gaps on efforts to prevent child marriage

There is a growing body of literature on efforts to prevent child marriage, or at least delay the age of marriage among girls. We have some evidence about “what works” from relatively small-scale and time-limited research studies and evaluated projects/programmes [9, 25–28], which acknowledge the need to respond to different drivers in different settings. However, there is much to learn, about scaling up programmes [25]. These include – the essential components to scale up, the required intensity and duration of implementation and the cost of scaling up. It also includes mechanisms to deliver them. In terms of effects, the questions include – the sustainability of changes in child marriage norms and practices, and the wider benefits of these changes on girls’ and women’s lives.

A lot of effort has gone in advocating for the formulation of laws specifying a minimum age for marriage. But little attention has gone into the application of these laws. Further research is needed to understand what works to effectively implement relevant laws. Research is also needed on the pros and cons of sanctions versus incentives [29–31].

As child marriage is underpinned by the same entrenched social and cultural norms which drive other attitudes and behaviors in communities, lessons may be drawn from the evidence-base for related behavior change approaches, for example, what has worked to shift other entrenched norms (e.g. around female genital mutilation/cutting, and safer sex strategies for HIV prevention in multiple settings).

Indicators of the age of marriage are useful to assess the effectiveness of efforts but they do not guide planners and implementers on the progress being made. Such indicators are urgently needed.

**Table:** 

Recommendations for further research	
•	Essential components of child marriage interventions to scale up, the required intensity and duration of implementation, mechanisms for delivering these interventions, and the cost of scaling up.
•	Sustainability of changes in child marriage norms and practices, and the wider benefits of these changes on girls’ and women’s lives.
•	Applying laws on child marriage.
•	Finding the right mix of sanctions and incentives.
•	Lessons that can be learned from other areas of social and cultural norm change.
•	Indicators that predict progress towards ending child marriage in different contexts.

### Research and evidence gaps on efforts to support child brides

Married girls can be difficult to access with information, services and programmes because of their social isolation and limited power within the household or community [32–34]. Targeted programming that engages them and the influential people around them e.g. husbands and mothers in law are required. There is a dearth of evidence about how best to support their needs.

There is also a need for further evidence on how to support the needs of girls who have escaped an unwanted marriage or have become widowed who often face abandonment and stigmatization.

**Table:** 

Recommendations for further research	
•	Levels of access of married girls to health, education and social services, and improving their access to and use of these services.
•	Links of married girls to community networks and resources, and enabling them to link up to and draw from them.
•	Supporting the development of equitable relationships between married girls and their (often older) husbands.
•	Needs of separated, divorced or widowed girls, and how to respond to them.

## Conclusions

There is significant evidence of the global scale and devastating impact of child marriage, demonstrating the need for greater investment in efforts to end child marriage. There are also increasing efforts to share lessons learned from projects and programmes that are under way. However, progress needs to be accelerated and results must be achieved on a much wider scale than what has been seen to date. To strengthen policy formulation and financial support to address child marriage, and to ensure that efforts are focused most effectively, further research will be crucial. Research efforts will also need to be better coordinated to strengthen collective learning. Agreeing on a set of global research priorities and a consistent set of outcome measures is important [8]. In addition, research to shape and guide policies and programmes at the national and subnational levels are also needed. In light of the target to end child marriage in the draft sustainable development goals and the impact that this will have on national development planning, we have both an opportunity and a responsibility to step up efforts to build the evidence base in this area.
